# City-scale resistome-mobilome architecture and mobility-associated ARG backbones across a megacity watershed

**DOI:** 10.1016/j.isci.2026.116841

**Published:** 2026-07-16

**Authors:** Ningning Pi, Xiaoyao He, Lu Zhu, Xinjue Hou, Xiaoyan Wu, Juan Zhang, Lili Yang, Dongjun Shen, Zhifen Zou, Rong Xiang, Xuan Wu

**Affiliations:** 1 College of Environment and Ecology, Chongqing University, 174 Shazheng Street, Chongqing 400044, China; 2 Key Laboratory of Environmental Chemistry and Ecotoxicology of Organic Pollutants of Chongqing, Chongqing Ecological and Environment Monitoring Center, 252 Qishan Road, Chongqing 401132, China; 3 Precision Medicine Center, The Second Affiliated Hospital of Chongqing Medical University, 288 Tianwen Road, Chongqing 400010, China; 4 Western (Chongqing) Institute for Digital-Intelligent Medicine, Chongqing National Biomedicine Industry Park, No. 28 Gaoxin Avenue, High-tech Zone, Chongqing 401329, China; 5 School of Traditional Chinese Medicine, Chongqing Medical University, Chongging 400016, China

**Keywords:** shotgun metagenomics, antimicrobial resistance, mobile genetic elements, co-selection, urban watershed

## Abstract

Antimicrobial resistance (AMR) in urban watersheds is shaped by diverse anthropogenic inputs, and linking reads-level resistome-mobilome associations to local antibiotic resistance gene (ARG) genetic contexts can strengthen environmental surveillance. Here, we analyzed 63 deeply sequenced shotgun metagenomes from a Chongqing megacity watershed spanning surface water, river sediments, wastewater treatment activated sludge, livestock wastewater, and hospital wastewater. Reads-based profiling revealed strong habitat structuring of ARG subtypes and MobileElementFinder-derived mobilome families, and total ARG loads co-varied with mobile genetic element (MGE), biocide resistance, and metal resistance axes. To add sequence-resolved context, we surveyed 8,043 ARG-carrying contigs and integrated element-level MGE calls with open reading frame (ORF)-level mobility functions to define putative mobility tiers. Wastewater-impacted habitats showed higher representation of contigs carrying conjugation-related mobility signals, whereas sediments exhibited high ARG and MGE loads but weaker ARG-MGE coupling. This megacity-scale framework prioritizes mobility-associated and co-selection genetic contexts for environmental AMR monitoring and mitigation.

## Highlights

• Dense megacity sampling reveals habitat-structured resistome-mobilome patterns
• Networks resolve ARG coupling into mobilization and co-selection axes
• 8,043 ARG-carrying contigs reveal genetic context and mobility tiers
• Wastewater habitats enrich ARG contigs with conjugation-related signals

## Graphical abstract

**Figure:**
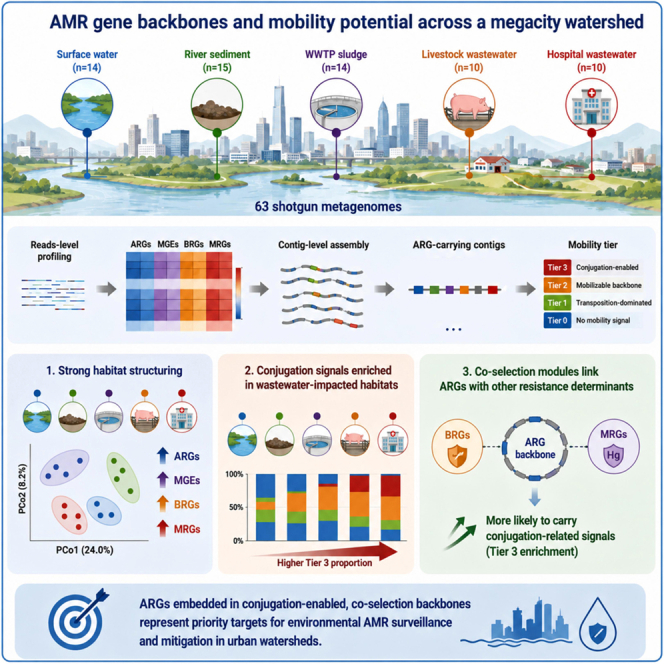

## Teaser

Health sciences; Public health; Environmental science

## Introduction

Antimicrobial resistance (AMR) is widely recognized as a One Health crisis linking clinical outcomes with environmental reservoirs and transmission pathways.^1^^,^^2^^,^^3^ While clinical surveillance tracks resistant pathogens, the environmental resistome—the collection of antibiotic resistance genes (ARGs) circulating in natural and engineered ecosystems—plays a critical role in maintaining and redistributing resistance determinants across human, animal, and environmental interfaces.^2^ Rivers and wastewater systems are particularly important because they integrate diverse anthropogenic inputs and provide routes for dissemination via water use, irrigation, and recreation.

Shotgun metagenomics has transformed AMR surveillance by enabling hypothesis-free profiling of ARGs and associated genetic determinants across complex microbial communities.^4^ Compared with targeted PCR panels, metagenomics captures a broader spectrum of resistance determinants and can simultaneously characterize co-occurring selective agents and mobility-associated genes. However, two gaps limit translation of metagenomic resistome surveys into actionable risk assessment. First, many studies provide broad descriptions of ARG abundance and composition but lack local depth across multiple habitats within a single watershed, making it difficult to distinguish stable habitat structuring from sampling noise. Second, when dissemination is evaluated primarily from reads-level abundance associations across samples, the resulting correlations can reflect shared sources or selection regimes rather than physical linkage on the same genetic element.^5^^,^^6^

A mechanistic understanding of dissemination requires attention to the mobile genetic elements (MGEs) that mediate horizontal gene transfer. Integrons, insertion sequences (ISs), transposons (Tn), plasmids, and integrative and conjugative elements (ICEs) are central vehicles of ARG acquisition and spread.^7^ Wastewater environments, characterized by high microbial density and diverse stressors, are often considered hotspots where MGEs and ARGs interact and where selection and horizontal transfer can shape resistome trajectories.^8^^,^^9^^,^^10^ Yet the mobilization potential of ARGs is heterogeneous: many ARGs occur as transposition-dominated cargo units, while others are embedded within conjugation-enabled backbones that plausibly support dissemination across hosts and habitats. Therefore, surveillance approaches that collapse all ARGs into a single abundance metric may miss the subset of ARGs carried in mobility-associated genetic contexts.

In addition to antibiotics, metals and biocides can co-select for antibiotic resistance because tolerance determinants and ARGs frequently co-occur on the same MGEs or plasmids.^11^^,^^12^^,^^13^ This is particularly relevant for urban watersheds where municipal wastewater, livestock discharges, and hospital effluents introduce mixed chemical stressors and microbial populations. Nonetheless, the extent to which co-selection manifests as contig-resolved module architectures—and whether such modules are more likely to be transfer-ready—remains underexplored in most city-scale river studies.

Here, we analyzed 63 deeply sequenced metagenomes across five habitats in a Chongqing megacity watershed (surface water [SW], river sediment [RSD], wastewater treatment plant [WT], livestock wastewater [LS], and hospital wastewater [HW]), providing dense within-city replication that complements global baselines with limited local depth.^14^ Rather than aiming for broad geographic coverage, this study was designed as a regionally focused, high-depth assessment within a single complex megacity watershed, where municipal wastewater treatment systems, LS, HW, SW, and sediments represent locally relevant environmental and anthropogenic compartments. We integrated habitat-resolved resistome and mobilome profiling; feature-level ARG coupling with MGE-, biocide resistance gene (BRG)-, and metal resistance gene (MRG)-associated axes; and contig-resolved genetic-context analysis of ARG-carrying sequences within the same city-scale framework. By integrating element-level MGE calls with open reading frame (ORF)-level mobility functions,^15^^,^^16^ we further stratified ARG-carrying contigs by mobility-associated evidence tiers and mapped non-antibiotic co-selection-associated neighborhoods, providing a regional analytical framework and data basis for interpreting within-watershed AMR structure and informing future locally tailored surveillance studies.

## Results

### Overview of resistome and mobilome across habitats

**Figure 1:**
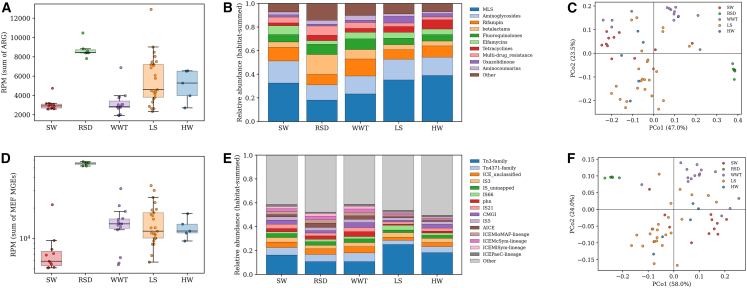
Overview of the resistome and mobilome across habitats (reads level) (A) ARG abundance per sample (RPM; sum across all ARG features), shown as boxplots with overlaid points (individual samples). (B) Habitat-summed ARG class composition. Stacked bars show relative abundance (habitat-summed RPM normalized to 1) for the top 10 ARG classes; remaining classes are grouped as “Other”. (C) PCoA of ARG profiles using Bray-Curtis dissimilarity on log1p (RPM). Each point represents one sample; axis labels report variance explained. (D) Total MGE abundance per sample quantified from MEF element IDs (RPM; sum across MEF elements). (E) Habitat-summed MEF MGE family composition. Stacked bars show relative abundance (habitat-summed RPM normalized to 1) for the top 15 MEF MGE families; remaining families are grouped as “Other”. (F) PCoA of MEF MGE family profiles using Bray-Curtis dissimilarity on log1p (RPM). SW, surface water; RSD, river sediment; WT, wastewater treatment plant; LS, livestock wastewater; HW, hospital wastewater. Habitat sample sizes are SW, *n* = 11; RSD, *n* = 7; WT, *n* = 15; LS, *n* = 25; and HW, *n* = 5. For boxplots in (A) and (D), data are represented by boxes showing the median, interquartile range, and 1.5× interquartile-range whiskers; points denote individual samples. Group-wise summaries and PERMANOVA outputs are provided in Tables S1–S3.

We profiled resistome and mobilome signatures across 63 metagenomes spanning five habitat types (SW, RSD, WT, LS, and HW). Total ARG burdens differed significantly across habitats (Kruskal-Wallis, *p* < 0.001; Table S1), with RSDs showing the highest overall ARG loads (mean 8,716.06 reads per million [RPM]), followed by LS (5,646.97 RPM) and HW (5,005.55 RPM), whereas SW exhibited the lowest ARG burdens (3,047.29 RPM) (Figure 1A; Table S2).

Across all samples, we detected 803 ARG subtypes spanning 28 antibiotic classes. The resistome was dominated by macrolide-lincosamide-streptogramin (MLS), aminoglycoside, rifampin, β-lactam, and fluoroquinolone resistance classes, which together accounted for the majority of ARG signals (Figures 1B and S2). To further connect the detected resistome with potential human-health relevance, we annotated drug-related ARG features according to the published risk-ranking framework described earlier (Table S4). Among the 803 drug-only ARG subtype labels, 59 could be assigned to the higher priority rank I–II categories, accounting for 16.22% of total drug-only ARG abundance. When drug-related multi-compound entries were also included, 67 labels were assigned to rank I–II, contributing 14.46% of total drug-related abundance. At the habitat level, cumulative rank I–II ARG abundance was highest in LS and RSDs, whereas SW showed the lowest relative contribution. These results provide an additional prioritization layer linking environmental ARG profiles to potential human-health relevance, without implying direct clinical risk from environmental detection alone.

Ordination of Bray-Curtis dissimilarities based on log1p-transformed ARG profiles revealed clear habitat structuring (Figure 1C), and a one-way permutational multivariate analysis of variance (PERMANOVA) confirmed a strong habitat effect on ARG composition (pseudo-F = 16.39, R^2^ = 0.531, *p* = 0.001; Table S3).

For the mobilome, MGEs were quantified from MobileElementFinder element IDs and summarized at the family level for downstream analyses. MGE loads were markedly elevated in sediments (mean 55,116.61 RPM), substantially exceeding those in other habitats (means 7,780–14,787 RPM) (Figure 1D; Table S2), and differed significantly across habitats (Kruskal-Wallis, *p* < 0.001; Table S1). Both MGE family composition (Figures 1E and S3) and principal coordinate analysis (PCoA) ordination (Figure 1F) showed pronounced habitat differentiation, supported by PERMANOVA (pseudo-F = 19.33, R^2^ = 0.571, *p* = 0.001; Table S3). Across gene categories, habitat explained the largest fraction of variance in MGE family composition (R^2^ = 0.571), followed closely by ARG subtypes (R^2^ = 0.531), whereas BRG and MRG profiles showed more moderate but still significant structuring (R^2^ = 0.397–0.411; Table S3). BRG/MRG and MULTI also displayed significant habitat dependence (Figure S4; Table S3).

### Dissecting resistome-mobilome coupling into mobilization and co-selection axes

**Figure 2:**
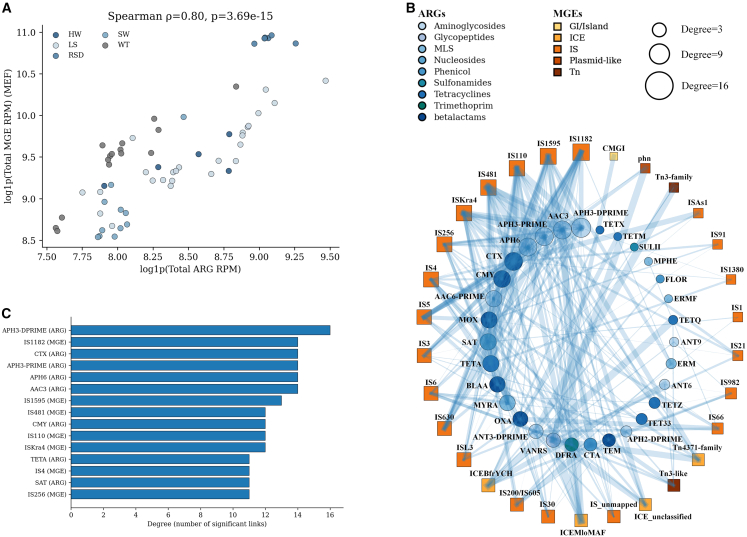
Reads-level ARG-MGE coupling and correlation network (A) Sample-wise relationship between total ARG abundance and total MEF MGE abundance. Points denote samples colored by habitat; axes are log1p (total RPM). Spearman’s ρ and *p* value are shown. (B) ARG-MGE correlation network inferred from reads-level abundance profiles. Circular nodes represent ARG features (colored by ARG class), and square nodes represent MEF MGE families (colored by major MGE type). Edges indicate significant Spearman correlations across samples (|ρ| ≥ 0.60, Benjamini-Hochberg false discovery rate [FDR] *q* < 0.05); edge width scales with |ρ|. Node size scales with degree (number of significant links). (C) Top hub nodes in the ARG-MGE network ranked by degree (top 15).

Given the pronounced habitat structuring of both resistome and mobilome profiles (Figures 1A–1F), we next asked whether cross-sample variation in ARG burden is primarily coupled to (1) mobilization potential (MGEs) and/or (2) co-selection determinants (biocide- and metal resistance). Across all samples, total ARG loads were strongly correlated with total MEF Element_ID-based MGE signals (Spearman ρ = 0.80, *p* = 3.69 × 10^−15^, *q* = 1.11 × 10^−14^; Figure 2A; Table S5). Habitat-specific correlation analysis further showed that ARG-MGE coupling was strongest in wastewater-impacted matrices, particularly LS (ρ = 0.90, *q* = 1.96 × 10^−9^) and wastewater treatment samples (WT: ρ = 0.87, *q* = 2.75 × 10^−5^). In contrast, RSDs showed no significant ARG-MGE coupling despite their high absolute ARG and MGE loads (ρ = 0.32, *q* = 0.819), and weaker or non-significant associations were also observed in SW (ρ = 0.58, *q* = 0.142) and HW (ρ = 0.70, *q* = 0.427). This pattern suggests that ARG-MGE abundance coupling was more pronounced in engineered or source-impacted wastewater matrices, whereas sediments may act as a relatively stable reservoir with weaker cross-sample ARG-MGE co-variation.

To resolve this coupling at the feature level, we separately quantified Spearman correlations between ARG features and each of three genetic axes: MGE families, biocide resistance determinants, and metal resistance determinants. In the ARG-MGE network (Figure 2B), associations were dominated by IS-type MGE families, indicating that IS lineages constitute the primary mobilization-linked layer of ARG covariance across samples. Hub inspection (Figure 2C) highlighted multiple IS families (e.g., IS1182, IS1595, IS481, and IS110) together with clinically relevant ARG features—particularly aminoglycoside and β-lactam determinants (e.g., APH3-DPRIME and CTX)—suggesting a shared, high-connectivity mobilization backbone supporting multi-class ARG distributions.

**Figure 3:**
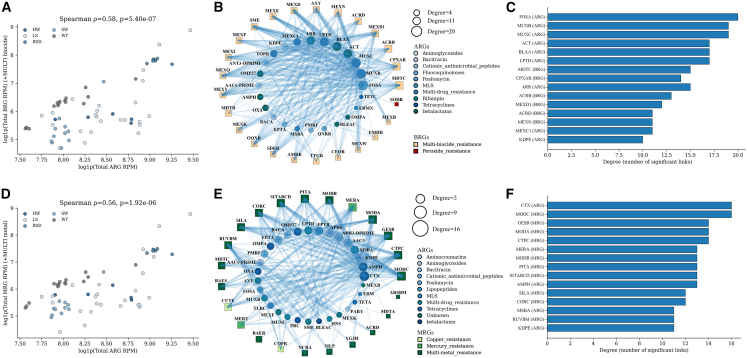
ARG co-variation with biocide and metal resistance determinants and corresponding networks (A) Sample-wise relationship between total ARG abundance and total BRG abundance. Axes are log1p (total RPM); points are colored by habitat; Spearman’s ρ and *p* value are shown. (B) ARG-BRG correlation network inferred from reads-level abundance profiles. Circular nodes represent ARG features (colored by ARG class), and square nodes represent BRG categories (colored by BRG type). Edges indicate significant Spearman correlations (|ρ| ≥ 0.60, BH FDR *q* < 0.05); edge width scales with |ρ|. Node size scales with degree. (C) Top hub nodes in the ARG-BRG network ranked by degree (top 15). (D) Sample-wise relationship between total ARG abundance and total MRG abundance. Axes are log1p (total RPM); points are colored by habitat; Spearman’s ρ and *p* value are shown. (E) ARG-MRG correlation network inferred from reads-level abundance profiles. Circular nodes represent ARG features (colored by ARG class), and square nodes represent MRG categories (colored by metal resistance type). Edges indicate significant Spearman correlations (|ρ| ≥ 0.60, BH FDR *q* < 0.05); edge width scales with |ρ|. Node size scales with degree. (F) Top hub nodes in the ARG-MRG network ranked by degree (top 15).

Beyond mobilization, total ARG loads also co-varied with the biocide resistance axis (ρ = 0.58, *p* = 5.40 × 10^−7^; Figure 3A; Table S5). The ARG-BRG network (Figure 3B) was structured around broad-spectrum biocide tolerance determinants and multi-biocide/multi-drug-associated features, forming dense positive associations with clinically important ARG features. Hub patterns (Figure 3C) highlighted ARG-side connectors, such as FOSA and multidrug resistance features (e.g., MUXB/MUXC), together with high-connectivity BRG-side nodes (e.g., MDTC and CPXAR), supporting a co-selection axis in wastewater-impacted settings.

Similarly, total ARG burdens correlated with the metal resistance axis (ρ = 0.54, *p* = 4.65 × 10^−6^; Figure 3D; Table S5). The ARG-MRG network (Figure 3E) emphasized multi-metal resistance modules and specific metal-associated markers (e.g., MERA and copper-related determinants) as recurrent connectors to ARG features. Consistent with this, hub patterns (Figure 3F) identified multiple MRG-side high-connectivity nodes (e.g., MODC and MERA) and ARG-side β-lactam determinants (e.g., CTX) as central elements, indicating that a subset of ARG variation tracks metal-associated resistance modules across samples.

### Contig-resolved evidence supports mobilization- and co-selection-linked associations

To complement cross-sample co-variation with sequence-resolved context, we performed a comprehensive contig-level survey across 63 metagenomes, yielding 8,043 ARG-carrying contigs. MobileElementFinder detected element-level MGE signatures on 411 contigs (Table S6). Among these, 386 contigs formed at least one ARG-MGE proximity record that could be mapped to 27 curated MGE families (Table S7), providing a contig-resolved counterpart to the reads-level ARG-MGE axis. In parallel, mobileOG identified mobility-related ORFs on 1,998 contigs (Table S6), supporting a complementary functional view of mobilization potential. Co-selective determinants were common on ARG-bearing backbones: 2,085 contigs showed ARG proximity to BRG-like determinants and 214 contigs to MRG-like determinants (Tables S8 and S9), noting that many of these links involved broad-spectrum efflux/regulatory determinants that can carry overlapping resistance annotations. Additional neighborhood examples are provided in Figures S5, S6, and S7. These records were therefore treated as conservative, contig-level proximity evidence and were not interpreted as direct evidence of complete mobile-element reconstruction, active horizontal transfer, or functional co-selection.

**Figure 4:**
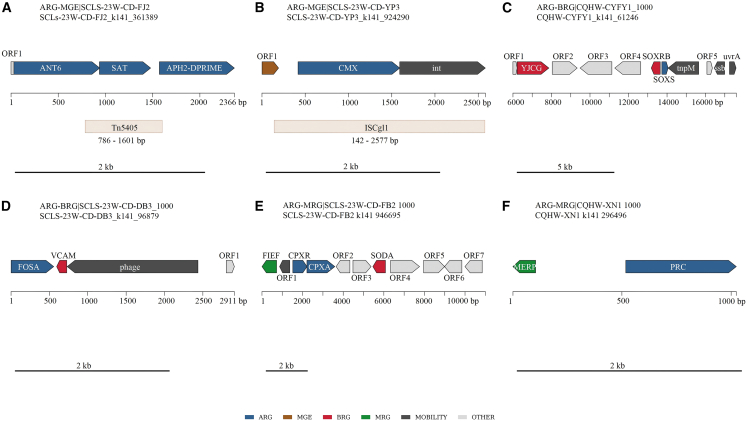
Representative contig-level genetic neighborhoods showing ARG proximity to MGE-, BRG-, or MRG-associated features (A) ARGs located adjacent to a Tn5405-associated region. (B) The phenicol resistance gene CMX located within an ISCgl1-associated region. (C) ARG- and BRG-associated features located together with mobility-associated ORFs. (D) ARG- and BRG-associated features located together with a phage-related ORF. (E) ARG-, MRG-, BRG-, and mobility-associated features located in the same gene neighborhood. (F) ARG- and MRG-associated features located in the same gene neighborhood. Arrows denote predicted ORFs oriented by strand. Shaded blocks denote MEF-called element regions. Scale bars are shown in each image. Colors denote feature categories as indicated in the legend.

Across ARG-MGE co-localization records (Table S7), ARGs most frequently occurred within IS- and Tn-associated contexts, consistent with an IS/Tn-dominated mobilization backbone. The most common MGE families proximal to ARGs included Tn3-family, IS6, IS1595, IS91, IS1380, and IS481 (Figure S8; Table S7), matching the prominent MGE families/types that structure the ARG-MGE correlation network (Figure 2B). Representative examples illustrate local ARG-MGE proximity on assembled contigs: a Tn3-family unit transposon (Tn5405) overlapped or was adjacent to a compact aminoglycoside resistance cassette (e.g., ANT6, SAT, and APH2-DPRIME) on an ARG-carrying contig (Figure 4A; Table S7), and an ISCgl1-associated segment encompassed CMX on a separate contig (Figure 4B; Table S7). Additional IS/Tn-centered neighborhoods supporting ARG-MGE linkage are shown in Figure S5, with complete contig-level records provided in Table S7. Together, these contig-resolved structures provide sequence-resolved support consistent with the mobilization axis inferred from reads-level correlations (Figures 2B and 2C), showing that a substantial fraction of high-connectivity ARGs can occur within or immediately adjacent to MGE-associated regions.

Contig-level BRG-like signals were widespread among ARG-carrying contigs, but their genetic contexts were dominated by broad-spectrum efflux systems and regulators rather than narrow biocide-specific enzymes (Figure S9; Table S8). The most frequent BRG-like categories proximal to ARGs were drug/biocide RND efflux pumps, SMR efflux pumps, and RND efflux regulators (Figure S9; Table S8), aligning with the BRG-side hubs and connectors observed in the ARG-BRG network (Figures 3B and 3C). A representative contig illustrates this co-selection architecture, where a SOXS-centered regulatory module co-occurred with ARGs in a compact neighborhood consistent with dual relevance to antibiotic and biocide tolerance (Figure 4C; Table S8). In another contig, FOSA occurred in close proximity to an ABC-type efflux component (VCAM) annotated as a multi-biocide efflux module (Figure 4D; Table S8). Additional ARG-BRG-like neighborhoods are provided in Figure S6, and all contig-level co-localization records are listed in Table S8. Collectively, these patterns suggest that the BRG-linked axis detected at the community level (Figures 3A–3C) often had contig-level counterparts in ARG adjacency to broad-spectrum efflux/regulatory modules, providing genetic-context evidence consistent with potential co-selection in wastewater-impacted environments.

Compared to biocide-linked determinants, metal-linked co-localization on ARG-carrying contigs was less frequent but revealed distinctive, interpretable cassettes (Table S9). The most common MRG-like categories near ARGs included multi-metal resistance proteins and metal-associated efflux/regulatory signatures, with notable contributions from copper- and mercury-associated modules (Figure S10; Table S9). Two representative neighborhoods illustrate these contexts: CPXAR occurred within ∼1–3 kb of the metal efflux component FIEF (Figure 4E; Table S9), and PRC was closely linked to the mercury resistance protein MERP (Figure 4F; Table S9). Additional ARG-MRG-like neighborhoods are shown in Figure S7, with complete contig-level records in Table S9. These contig-resolved observations provide a concrete structural complement to the ARG-MRG correlation network (Figures 3E and 3F), indicating that at least a subset of metal-linked covariance can be traced to co-localized resistance modules on ARG-bearing genetic backbones.

Together, the contig survey reveals a coherent hierarchy of local genetic contexts that is consistent with the three correlation axes established in Section dissecting resistome-mobilome coupling into mobilization and co-selection axes. The mobilization axis is most directly supported by contig architectures in which ARGs reside within IS/Tn-associated regions (Figures 4A, 4B, and S5; Table S7), consistent with the IS/Tn-dominated structure of the ARG-MGE network (Figures 2B and 2C). In parallel, co-selection signatures are frequently realized through proximity to broad-spectrum efflux/regulatory modules (Figures 4C, 4D, and S6; Table S8) and, less commonly, through recognizable metal resistance cassettes (Figures 4E, 4F, and S7; Table S9), providing genetic context for the corresponding reads-level networks (Figures 3B, 3C, 3E, and 3F). These convergent lines of evidence support a cautious interpretation that some strong cross-sample associations may reflect biologically plausible genetic organizations, while the broader diversity of local proximity patterns captured by the contig survey is documented in the supplementary material (Figures S5, S6, S7, S8, S9, and S10; Tables S6, S7, S8, and S9).

### MEF-mobileOG integration highlights mobility potential beyond local proximity

Building on contig-level observations showing that a subset of reads-level associations is consistent with sequence proximity, we next asked which ARG-bearing contigs contained mobility-associated genetic features compatible with mobilization potential.

Across 8,043 ARG-carrying contigs, MEF detected conservative element-level MGE signals on 411 contigs (5.1%), whereas mobileOG annotated mobility functions on 1,998 contigs (24.8%) (Table S6). Importantly, these signals only partially overlapped: 116 contigs carried both MEF-detected MGEs and mobileOG mobility ORFs, while 295 were MEF-positive but mobileOG-negative and 1,882 were mobileOG-positive but MEF-negative (Figure S11). This complementarity suggests that element boundary calling (MEF) and mobility-module annotation (mobileOG) capture distinct layers of mobilization architecture.

**Figure 5:**
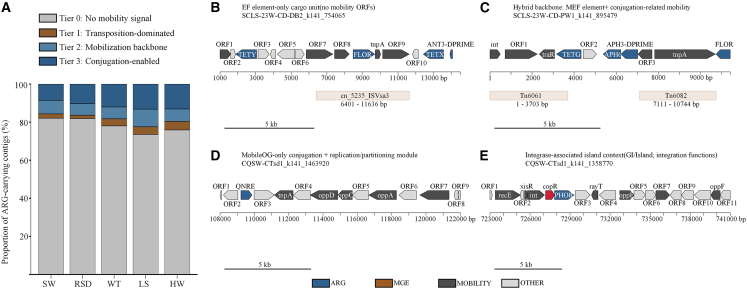
Representative ARG-carrying contig contexts with different layers of mobility-associated evidence (A) Tier composition across habitats among ARG-carrying contigs, based on the MEF-mobileOG tiering scheme defined in STAR Methods. (B) A composite-transposon-associated ARG locus with MEF-called element regions and mobileOG-annotated ORFs. (C) A Tn-family-associated ARG locus with MEF-called element regions and mobileOG-annotated ORFs. (D) An ARG-containing mobileOG-only locus without an explicit MEF-called element region. (E) An ARG-containing integration-associated locus with mobileOG-annotated ORFs. Arrows denote predicted ORFs oriented by strand. Shaded blocks denote MEF-called element regions. Scale bars are shown in each image. Colors denote feature categories as indicated in the legend.

To translate this two-layer annotation into an interpretable mobilization gradient, we stratified ARG-carrying contigs into mobilization tiers, from no detectable mobility signal (tier 0) to conjugation-enabled contexts (tier 3). Tier composition differed systematically across habitats: tier 3 increased from 8.5%–10.3% in SW/RSD to 12.0%–13.1% in WT/LS/HW (Figure 5A), suggesting that wastewater-impacted environments were enriched in ARG-carrying contigs with conjugation-related mobility signals. In parallel, MEF-positive contigs were more frequent in WT/LS/HW (∼5.4%–5.8%) than in SW/RSD (∼2.3%) (Figure S12), reinforcing the habitat structuring of mobilization signals.

Representative contig architectures illustrate the tier concept (Figures 5B–5E). We observed (1) MEF-detected element-associated ARG cargo regions carrying transposition/integration-related mobility signals but lacking complete conjugation/relaxase modules on the same contig (Figure 5B; see also IS/Tn cargo examples in Figures 4A and 4B); (2) hybrid contexts where MEF-called elements co-occur with conjugation-related mobility functions (Figure 5C); (3) mobileOG-only regions containing transfer- or maintenance-associated ORFs without explicit MEF element boundaries, indicating mobility-associated signals that may be missed by conservative element calling (Figure 5D); and (4) integration-associated local contexts consistent with island-like mobility features (Figure 5E). Collectively, these architectures provide a conceptual bridge between reads-level ARG-MGE associations and contig-resolved transfer readiness.

Finally, linking to the reads-level network framework, hub ARG features showed a non-random distribution across tiers: hub ARGs were enriched in transposition-dominated contexts, whereas conjugation modules represented an additional mobility-associated layer in specific habitats (Figure S13).

To further extend the human-health-oriented interpretation of ARG-carrying contigs, we performed a taxonomic-context analysis. Among 8,043 ARG-carrying contigs, 7,897 contigs (98.18%) received a taxonomic assignment, and 4,936 contigs (61.37%) were classified at the genus or species level. Using a curated list of taxa containing opportunistic, enteric, waterborne, or human-associated pathogenic members, 2,328 ARG-carrying contigs (28.94%) were conservatively assigned to potential pathogen-associated taxa. These candidates were mainly detected in LS (*n* = 1,246) and HW (*n* = 445), followed by RSDs (*n* = 316), SW (*n* = 186), and wastewater treatment samples (*n* = 135). The most frequent potential pathogen-associated genera included *Pseudomonas*, *Acinetobacter*, *Escherichia*, *Clostridium*, *Citrobacter*, *Enterococcus*, *Streptococcus*, and *Klebsiella* (Table S10). Most pathogen-associated candidates occurred in tier 0–1 contexts, whereas 109 candidates were assigned to tier 2–3 mobility-associated contexts. These results provide an additional host-context prioritization layer for ARGs with potential human-health relevance, while not implying confirmed pathogenic hosts or direct clinical risk from environmental detection alone.

### Co-selection-associated neighborhoods on ARG-carrying contigs: BRG/MRG coupling and putative mobility stratification

**Figure 6:**
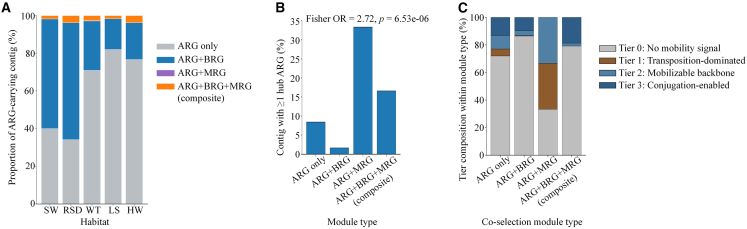
Co-selection-associated neighborhoods on ARG-carrying contigs integrate BRG/MRG coupling with putative mobility tiers (A) Co-selection-associated neighborhood classes across habitats. ARG-carrying contigs were classified into mutually exclusive module types based on the presence of BRG and/or MRG within a ±5 kb neighborhood window around ARG features (ARG-only, ARG+BRG-only, ARG+MRG-only, and composite ARG+BRG+MRG). Stacked bars show the proportion of module types within each habitat. (B) Hub ARG enrichment by neighborhood type. Bars show the percentage of contigs in each module class that contain ≥1 hub ARG features (hub definition derived from reads-level correlation networks); Fisher’s exact test statistics are shown. (C) Putative mobility-tier composition within each neighborhood type. Stacked bars show the distribution of the four mobilization tiers across module classes. Fisher’s exact tests with Benjamini-Hochberg correction were used for enrichment analyses.

To evaluate how non-antibiotic stressors may shape contig-resolved co-selection neighborhoods, we classified ARG-carrying contigs into mutually exclusive module classes using a ±5 kb window around ARG ORFs: ARG-only (no BRG/MRG in the window), ARG+BRG-only (BRG present but no MRG), ARG+MRG-only (MRG present but no BRG), and composite (ARG+BRG+MRG). This yielded 5,958 ARG-only contigs, 1,895 ARG+BRG-only contigs, 3 ARG+MRG-only contigs, and 187 composite contigs (Table S11). When counting composite contigs within both axes, 2,082 contigs involved BRG and 190 involved MRG (Figure 6A), indicating that BRG/MRG-associated ARG neighborhoods were not limited to isolated events but recurred as interpretable neighborhood classes that can be compared across environments.

Architecture analysis indicated that ARG+BRG-only modules were dominated by broad-spectrum efflux and regulatory configurations, whereas MRG-involving neighborhoods more often formed transport-centered multi-metal cassettes (Figures S6 and S7).

Integrating putative mobility tiers further revealed that co-selection-associated neighborhood classes differed in their mobility-associated signals. Composite modules showed a significantly higher likelihood of carrying conjugation-related tier 3 signals compared with non-composite contigs (odds ratio [OR] = 1.63, *p* = 0.013; Table S12). In contrast, BRG-involving contigs were overall depleted for tier 3 contexts (OR = 0.77, *p* = 0.0012; Table S12), whereas MRG-involving contigs were enriched in tier 3 (OR = 1.60, *p* = 0.019; Table S12). Consistent with these statistics, tier composition profiles differed by mutually exclusive module class (Figure 6C; Table S11): tier 3 accounted for 18.7% of composite modules, compared with 13.2% of ARG-only contigs and 9.7% of ARG+BRG-only modules. Together, these results suggest a contextual partitioning in which biocide-linked ARG neighborhoods were common but not necessarily enriched in tier 3 mobility signals, whereas composite and MRG-involving architectures more frequently aligned with conjugation-related contig contexts under mixed stressor regimes. Composite modules were enriched for contigs carrying ≥1 hub ARG feature (Fisher OR = 2.72, *p* = 6.53 × 10^−6^; Figure 6B; Table S13).

## Discussion

AMR in aquatic environments has been intensively profiled by metagenomics, yet two persistent challenges remain: (1) resolving habitat-specific AMR “architecture” within a single urban watershed, where multiple anthropogenic sources co-occur, and (2) moving beyond abundance correlations to infer plausible dissemination potential.^2^^,^^4^ By densely sampling a major megacity watershed and integrating reads-level profiling with contig-resolved genomic context, our study provides a context-resolved view of how ARGs are structured across habitats and how they are associated with mobility signals under multi-stressor urban conditions.

A consistent outcome across global and regional studies is that sediments function as persistent sinks of resistance determinants, often showing higher ARG load and a distinct community-structured resistome than the overlying water.^8^^,^^9^ Our data reinforce this pattern and extend it by showing that sediment-associated resistomes form a robust habitat-defined cluster, suggesting that sediment acts not only as a sink but as a distinct ecological compartment that integrates long-term inputs and promotes retention of resistance determinants. Mechanistically, this is consistent with the enhanced persistence of extracellular DNA and particle-associated genetic material in sediments, which can preserve plasmid-borne and mobile ARG fragments and facilitate transformation under favorable conditions.^17^ The implication is that interventions targeting only the water column (e.g., effluent polishing) may underestimate the legacy reservoir embedded in benthic matrices, which can be remobilized by hydrologic disturbance or sediment resuspension.

In our dataset, sediments also exhibited high MGE loads but weak reads-level ARG-MGE coupling, suggesting that heterogeneous inputs and long-term accumulation can decouple short-term abundance co-variation from the underlying mobilome reservoir. Together with the elevated transfer readiness observed in wastewater-impacted habitats, this pattern supports a reservoir-amplifier contrast in which sediments store long-lived resistance inventories, whereas engineered and effluent-connected systems concentrate transfer-capable backbones.

While habitat-level patterns are informative, interpreting reads-level abundance co-variation in terms of dissemination requires caution. Correlation networks can highlight co-varying determinants and generate testable hypotheses, yet they are sensitive to compositional effects, sparsity, and indirect associations.^5^^,^^6^^,^^18^ To strengthen interpretation, we complemented these networks with contig-resolved gene-neighborhood analysis that evaluates whether co-varying features also occur in close proximity on assembled sequences, particularly near MGE- or mobility-associated functions. Because two genes can co-vary across samples due to shared sources or selective regimes without residing on the same genetic vehicle, sequence-resolved proximity provides additional contextual support for prioritizing biologically plausible linkage patterns and helps prioritize candidates for downstream validation.^7^ These analyses were based on short-read assemblies; we interpret ARG-MGE, ARG-BRG, and ARG-MRG co-localization as local genetic-context evidence rather than direct proof of complete mobile elements, horizontal transfer, or functional co-selection.

Notably, a subset of highly connected ARG features in reads-level networks was supported by contig architectures that physically linked ARGs to IS/Tn- or ICE-associated contexts. This network-to-contig triangulation increases confidence that at least part of the observed ARG-MGE coupling reflects shared genetic backbones, rather than only shared ecological gradients.

Together with mobilization tiering, this network-to-contig-to-tier triangulation helps prioritize candidate backbones that are both epidemiologically connected (network hubs) and structurally consistent with mobilization, rather than reflecting shared ecological gradients alone.

To interpret dissemination potential more mechanistically, we integrated two complementary layers of mobility evidence: MEF’s conservative element-level calls and mobileOG’s ORF-level mobility functions.^15^^,^^16^ These tools answer different biological questions. MEF captures recognizable element boundaries and curated element IDs, whereas mobileOG can capture mobility-related ORFs even when element boundaries are ambiguous or fragmented by assembly. The partial overlap between MEF-positive contigs and contigs containing mobility ORFs in our dataset therefore likely reflects biological reality and technical complementarity: many ARGs reside in transposition-dominated “cargo” units (IS/Tn composites) without a full conjugation apparatus on the same contig, while other contigs contain mobility backbones (conjugation, replication/partitioning, and integration) that may not be assigned a classic element boundary by MEF.

This complementarity is not merely a methodological detail; it enables a more interpretable metric—mobilization tiering—that approximates “transfer readiness” beyond simple co-localization. Tiering also helped reveal habitat tendencies: wastewater-impacted habitats showed higher representation of conjugation-enabled contexts, consistent with long-standing views that wastewater systems can act as incubators for horizontal gene transfer under dense microbial contact and selective pressure.^8^^,^^9^^,^^10^ Importantly, this tier framework provides a practical monitoring concept: rather than tracking thousands of ARG features individually, surveillance can prioritize ARGs embedded in tier 2–3 contexts, which are more likely to disseminate via mobile backbones.

Environmental AMR is not governed by antibiotics alone; metals and biocides can co-select for resistance because resistance and tolerance determinants frequently co-occur on the same plasmids or composite MGEs.^11^^,^^12^^,^^13^ Our results support this paradigm but add nuance by separating co-selection into contig-defined module classes. ARG+BRG modules were abundant and widespread, consistent with the pervasive use of disinfectants and biocides in urban systems and animal production. In contrast, MRG-involving modules were less frequent but displayed a stronger association with higher mobilization tiers, and composite ARG+BRG+MRG modules were enriched in tier 3 conjugation-related signals. This pattern supports a mixed-stressor hypothesis: BRG coupling may be common and ecologically stabilizing, but when BRG and MRG co-occur with ARGs on the same contig, the architecture more often carries mobility-associated features consistent with higher putative mobilization potential.^7^^,^^13^ This apparent contrast likely reflects that the BRG axis is dominated by ubiquitous efflux/regulatory determinants that frequently occur on non-conjugative backbones, whereas MRG signals—though rarer—tend to be concentrated on conjugation-enabled architectures.^7^^,^^11^^,^^12^^,^^13^

One practical implication is that co-selection risk is not uniform across non-antibiotic stressors. Metals can exert persistent selection in sediments and sludge and can maintain plasmids carrying linked resistance determinants even when antibiotic concentrations are low.^11^ Biocides, particularly quaternary ammonium compound usage in healthcare and sanitation settings, may similarly favor determinants that couple tolerance and multidrug efflux with ARG cargo.^12^^,^^19^^,^^20^ Our contig-centric module framework provides a way to help differentiate ubiquitous co-occurrence from the subset of configurations most likely to disseminate.

A frequent limitation of global surveys is the trade-off between geographic breadth and local depth. For example, global sewage metagenomics provides invaluable cross-country comparisons but typically includes limited replication within any single city or watershed.^14^ By focusing on Chongqing—a major megacity with complex, densely connected wastewater and river networks—our design offers high within-region replication across multiple anthropogenic sources (municipal treatment, livestock, and hospitals) and environmental reservoirs (SW and sediments). This density improves statistical power for habitat comparisons and allows more confident inference of within-watershed structuring rather than conflating local variability with broad geographic gradients. In other words, our dataset complements global surveys: broad studies define global baselines, whereas deep city-scale sampling can identify locally actionable hotspots and dissemination architectures.

In addition to within-region replication, deep sequencing improved sensitivity for low-abundance determinants and increased recovery of ARG-carrying contigs, supporting more stable habitat comparisons and context analyses across contrasting matrices.

Overall, this study advances urban watershed AMR surveillance from descriptive profiling toward a more context-resolved interpretation. We integrated habitat-resolved resistome and mobilome profiles with correlation networks and contig-resolved genomic context to help differentiate ecological co-occurrence from contig-supported linkage patterns. This integration clarifies how ARG-MGE coupling observed at the community level can be supported by specific co-localization patterns and mobility contexts, and it extends co-selection analyses by explicitly mapping BRG- and MRG-associated modules on ARG-carrying contigs. By applying this framework within a densely sampled megacity system, the results provide a locally grounded view of where resistance reservoirs and ARG-carrying contigs with stronger mobility-associated signals are most likely to concentrate, offering a practical basis for prioritizing monitoring and mitigation in complex urban water networks.

### Limitations of the study

This study has several limitations. First, the genetic-context analysis was based on short-read metagenomic assemblies. Although ARG-carrying contig screening provided sequence-resolved local neighborhood information, short-read contigs cannot reliably reconstruct complete plasmids, ICEs, composite Tn, or host-linked mobile elements. Therefore, ARG-MGE, ARG-BRG, and ARG-MRG proximity patterns and putative mobility tiers should be interpreted as annotation-based evidence of local genetic context and mobility-associated potential, rather than as direct evidence of complete transferable elements, active horizontal gene transfer, or functional co-selection. Second, taxonomic assignments of ARG-carrying contigs were used only as a host-context prioritization layer and should not be interpreted as definitive pathogen-host assignment or direct clinical risk estimation. Third, this study focused on one megacity watershed, and broader geographic, seasonal, and temporal sampling will be needed to evaluate how generalizable these habitat-associated patterns are across other urban watershed systems. Future studies integrating long-read metagenomics, Hi-C, plasmidome sequencing, plasmidome profiling, single-cell genomics, and experimental validation would help resolve complete ARG-bearing mobile elements, host associations, and transfer potential.

## Resource availability

### Lead contact

Requests for further information and resources should be directed to and will be fulfilled by the lead contact, Xuan Wu (wuxuan329329@163.com).

### Materials availability

This study did not generate new unique reagents.

### Data and code availability

• Raw shotgun metagenomic sequencing data generated in this study have been deposited in the China National Center for Bioinformation under accession numbers CRA026854, CRA026878, CRA026940, CRA026943, and CRA026891 and are publicly available as of the date of publication.
• Processed summary tables are provided in Document S1, and large machine-readable supplemental tables are provided as Excel files for Tables S4, S6, S7, S8, S9, and S10.
• This paper does not report original code.
• Any additional information required to reanalyze the data reported in this paper is available from the lead contact upon request.

## Acknowledgments

This work was supported by the Chongqing Municipal Special Fund for Technological Innovation and Application Development (grant nos. CSTB2024TIAD-GPX0010 and CSTB2022TIAD-KPX0115), the 10.13039/501100001809National Natural Science Foundation of China (grant nos. 32060024 and 32000088), the Chongqing Municipal Social Science Planning Project (2025ZDZK27), and the Environmental Monitoring Research and Development Fund (CQHJ-NBKY-2024-003).

## Author contributions

Conceptualization, Xuan Wu and R.X.; methodology, N.P., X. He, L.Z., and Xuan Wu; investigation, N.P., X. He, L.Z., X. Hou, Xiaoyan Wu , J.Z., L.Y., D.S., and Z.Z.; formal analysis, N.P. and Xuan Wu; resources, Xuan Wu and R.X.; writing – original draft, N.P. and Xuan Wu; writing – review and editing, all authors; supervision, Xuan Wu and R.X.; funding acquisition, Xuan Wu.

## Declaration of interests

The authors declare no competing interests.

## STAR★Methods

### Key resources table

**Table:** 

REAGENT or RESOURCE	SOURCE	IDENTIFIER
**Biological samples**		

Surface water samples (SW, *n* = 11)	This paper	N/A
River sediment samples (RSD, *n* = 7)	This paper	N/A
Activated sludge samples from municipal wastewater treatment plants (WT, *n* = 15)	This paper	N/A
Livestock wastewater samples from intensive swine farms (LS, *n* = 25)	This paper	N/A
Hospital wastewater final effluent samples (HW, *n* = 5)	This paper	N/A

**Critical commercial assays**		

DNeasy PowerWater Kit	QIAGEN	Cat# 14900-100-NF
DNeasy PowerSoil Pro Kit	QIAGEN	Cat# 47016
Qubit dsDNA HS Assay Kit	Thermo Fisher Scientific	Cat# Q32854
NEBNext Ultra II DNA Library Prep Kit for Illumina	New England Biolabs	Cat# E7645L
AMPure XP beads	Beckman Coulter	Cat# A63882

**Deposited data**		

Raw shotgun metagenomic data from surface water samples	China National Center for Bioinformation (CNCB)	CNCB: CRA026854
Raw shotgun metagenomic data from river sediment samples	China National Center for Bioinformation (CNCB)	CNCB: CRA026878
Raw shotgun metagenomic data from wastewater treatment activated sludge samples	China National Center for Bioinformation (CNCB)	CNCB: CRA026940
Raw shotgun metagenomic data from livestock wastewater samples	China National Center for Bioinformation (CNCB)	CNCB: CRA026943
Raw shotgun metagenomic data from hospital wastewater samples	China National Center for Bioinformation (CNCB)	CNCB: CRA026891
Processed abundance, annotation, correlation, and contig-context tables	This paper	Tables S1–S3, S4, S5, S6, S7, S8, S9, S10, and S11–S13

**Software and algorithms**		

AMRPlusPlus v2.0	AMRPlusPlus project	https://github.com/Microbial-Ecology-Group/AMRplusplus
BWA-MEM v0.7.17	BWA project	https://github.com/lh3/bwa
MEGARes v3.0	MEGARes database	https://www.meglab.org/megares
BacMet v2.0	BacMet database	http://bacmet.biomedicine.gu.se
MobileElementFinder	Johansson et al.15; Center for Genomic Epidemiology	https://cge.food.dtu.dk/services/MobileElementFinder/
MEGAHIT	MEGAHIT project	https://github.com/voutcn/megahit
Prodigal	Prodigal project	https://github.com/hyattpd/Prodigal
DIAMOND	DIAMOND project	https://github.com/bbuchfink/diamond
mobileOG-db beatrix 1.6	Brown et al.16; mobileOG-db project	https://mobileogdb.flsi.cloud.vt.edu
Kraken2 with Standard database	Wood et al.18; Kraken2 project	https://github.com/DerrickWood/kraken2
R	R Project for Statistical Computing	https://www.r-project.org
Python	Python Software Foundation	https://www.python.org
vegan R package	vegan project	https://CRAN.R-project.org/package=vegan
SciPy	SciPy project	https://scipy.org
NumPy	NumPy project	https://numpy.org
pandas	pandas project	https://pandas.pydata.org
NetworkX v3.3	NetworkX project	https://networkx.org
Gephi v0.9.2	Gephi project	https://gephi.org

**Other**		

Stainless-steel Van Veen grab	Hydro-Bios	Product ID 437 330
0.22 μm polyethersulfone membrane filters	MilliporeSigma	Cat# GPWP04700
NanoDrop ND-2000 spectrophotometer	Thermo Fisher Scientific	N/A
Qubit 4 fluorometer	Thermo Fisher Scientific	N/A
Covaris DNA fragmentation instrument	Covaris	500217
Agilent 2100 Bioanalyzer	Agilent Technologies	N/A
Illumina NovaSeq 6000 sequencing platform	Illumina	N/A

### Experimental model and study participant details

This study used environmental samples only and did not involve human participants, patient-derived specimens, experimental animals, cell lines, or microbial isolates. Surface water (SW, *n* = 11) grab samples (20 L each) were taken at mid-channel stations along the mainstem of the Yangtze River in Chongqing with pre-sterilized high-density-polyethylene (HDPE) carboys rinsed twice with site water. River sediment (RSD, *n* = 7) samples (100 g per site) were collected with a stainless-steel Van Veen grab (Hydro-Bios, Germany); the surficial 0–5 cm layer was sampled and composited into sterile polypropylene jars.

Wastewater treatment plant samples were collected as activated sludge (WT, *n* = 15) from aeration tanks of fifteen municipal wastewater-treatment plants serving the Chongqing metropolitan area. Livestock wastewater (LS, *n* = 25) was collected from discharge outlets or settling ponds of intensive swine farms, and hospital wastewater (HW, *n* = 5) was collected as final effluents of tertiary-grade general hospitals. All sampling sites were located in Chongqing and were arranged within the Yangtze River watershed, with surface-water and sediment samples collected along the Yangtze River mainstem and pollution-source samples selected from municipal wastewater treatment plants, intensive swine farms, and tertiary hospitals that are hydrologically connected to the Yangtze River system through surface runoff, drainage networks, wastewater conveyance systems, or downstream receiving waters. The spatial distribution of all sampling sites is shown in Figure S1. All containers were kept on ice (<4°C) during transport and processed within 6 h of collection to minimize DNA degradation.

### Method details

#### DNA extraction and metagenomic sequencing

All 20 L of each aqueous sample (SW, LS, and HW) were vacuum-filtered through sterile 0.22 μm polyethersulfone membrane filters (47 mm diameter; MilliporeSigma). Filters were replaced whenever flow rates declined to ensure the entire volume was processed. Collected membranes were folded with sterile forceps, transferred to bead-beating tubes, and stored at −80°C until extraction. Genomic DNA was isolated with the DNeasy PowerWater Kit (Qiagen) following the manufacturer’s protocol.

For RSD and WT samples, 0.5 g (wet weight) of homogenized material was processed using the DNeasy PowerSoil Pro Kit (Qiagen) with bead-beating lysis. DNA purity and concentration were assessed with a NanoDrop ND-2000 spectrophotometer and a Qubit 4 fluorometer using the Qubit dsDNA HS Assay Kit.

Metagenomic libraries were constructed using the NEBNext Ultra II DNA Library Prep Kit for Illumina. Briefly, 1 μg DNA was fragmented to approximately 350 bp using a Covaris DNA fragmentation instrument, end-repaired, A-tailed, ligated to Illumina adapters, PCR-enriched, purified with AMPure XP beads, and quality-checked on an Agilent 2100 Bioanalyzer. Indexed libraries were pooled equimolarly and sequenced in paired-end 150 bp mode on an Illumina NovaSeq 6000 platform. Each sample generated ≥20 Gb of raw data.

#### Read-based profiling of resistome and co-selective determinants

After quality filtering, reads were mapped to reference gene catalogs to quantify antibiotic resistance genes (ARGs), mobile genetic elements (MGEs), metal-resistance genes (MRGs), biocide-resistance genes (BRGs), and multi-compound resistance determinants (MULTIs). Read mapping and post-filtering were implemented with AMRPlusPlus v2.0 configured to use BWA-MEM v0.7.17 (-k 31, -A 1, -B 4, -O 6, -E 1, -T 30). Alignments were retained only when covering ≥90% of the reference gene length and showing ≥95% sequence identity. ARGs were quantified against MEGARes v3.0; MRGs and BRGs were quantified against curated BacMet v2.0 subsets. MGE features were quantified by mapping reads to the MobileElementFinder element reference set (Element_IDs) and then summarized to family and major-type levels for downstream analyses.

For ecological reporting, each Element_ID was mapped to a standardized major MGE type and a family/lineage label. Minor MobileElementFinder categories were conservatively harmonized into major classes, including IME/CIME to ICE, MITE to Tn, and integron/island annotations to GI/Island. Type- and family-level profiles were obtained by summing reads per million (RPM) values across Element_IDs assigned to the same type or family. Abundances were normalized as RPM: RPM = (mapped reads × 10^6^)/total clean reads. RPM was used consistently across ARG, MGE, BRG, and MRG profiles to ensure direct comparability among gene categories. For reads-level summaries and correlation networks, MULTI determinants were reassigned into the BRG and/or MRG axes when their curated functional annotation indicated biocide- or metal-linked resistance/tolerance; determinants spanning both annotations contributed to both axes.

To provide an additional human-health-oriented prioritization layer, detected drug-related ARG features were further annotated using the published ARG risk-ranking framework of Zhang et al. In this analysis, MEGARes records assigned to the “Drugs” category and “Multi-compound” records containing drug-related annotations were included. Because MEGARes and SARG use different nomenclature systems, ARG features were conservatively mapped to published risk-ranked ARG families at the gene-family level where possible. Features that could not be confidently matched were retained as “Unmatched” rather than force-assigned. For each sample and habitat, RPM values were summed by risk-rank category. This analysis was used as a supplementary prioritization layer and was not interpreted as a direct quantitative estimate of human-health risk.

#### Assembly and contig-resolved resistance annotation

Quality-filtered reads were *de novo* assembled with MEGAHIT using default metagenomic settings, and contigs shorter than 1,000 bp were discarded. ORFs were predicted with Prodigal in metagenomic mode (-p meta). Predicted proteins were annotated by DIAMOND blastp searches against the MEGARes v3.0 protein reference to identify ARGs, retaining the best hit per ORF with e-value ≤1e−10, amino acid identity ≥80%, and query coverage ≥70%. Contigs containing ≥1 ARG ORF were defined as ARG-carrying contigs and retained for downstream context analyses. ORFs on ARG-carrying contigs were further screened for metal-, biocide-, and multi-compound resistance determinants (MRGs, BRGs, and MULTI) using curated BacMet v2.0 subsets and in-house resistance catalogs with the same DIAMOND-based criteria.

#### MGE and mobility annotation on ARG-carrying contigs

Element-level MGE regions on ARG-carrying contigs were predicted using MobileElementFinder (MEF; mefinder; mefinder find -c contigs.fasta -j -g -t), which reports element IDs, spans, and element types. For ecological reporting and for reads-level network nodes, MEF element IDs were aggregated to standardized major MGE types and family/lineage labels as described above. Family- and type-level MGE abundances were obtained by summing RPM across elements assigned to the same family or type.

To complement element calls, mobility- and transfer-related ORFs were annotated at the protein level using DIAMOND blastp against mobileOG-db (beatrix 1.6), retaining the best hit per ORF with e-value ≤1e−10 and --max-target-seqs 5. Mobility hits were grouped into conjugation/relaxase, replication/partitioning, integration/recombination, and transposition-related functions for summarization. Physical linkage on ARG-carrying contigs was assessed by integrating ORF coordinates and annotations; distances between feature midpoints were used to quantify ARG proximity to MEF MGE families and to co-selective determinants (BRG/MRG, including reassigned MULTI where applicable). Because resistance catalogs can overlap, particularly for broad-spectrum efflux pumps and regulators, cases where the same ORF carried dual annotations, such as ARG and BRG/MRG, were interpreted as overlapping or dual-function determinants contributing to the co-selection axis, rather than as two independently co-localized genes.

ARG-centered co-selection modules were defined using a ±5 kb window around each ARG ORF and assigned to mutually exclusive classes: ARG-only, with no BRG or MRG in the window; ARG+BRG-only, with BRG present and no MRG; ARG+MRG-only, with MRG present and no BRG; or composite ARG+BRG+MRG. Mobility-associated evidence was tiered by integrating MEF interval evidence with mobileOG functions: Tier 0, no MEF interval and no mobility ORFs; Tier 1, transposition-dominated, with IS/Tn signatures and/or transposition ORFs without conjugation/relaxase; Tier 2, mobilizable backbone, with replication/partitioning and/or integration/recombination without conjugation/relaxase; and Tier 3, conjugation-enabled, with any conjugation/relaxase marker, including type IV secretion system (T4SS), mating pair formation (MPF), or relaxase, regardless of other signals. Representative contigs were visualized as gene-neighborhood diagrams along contig coordinates, with ORFs color-coded by category and MEF spans shown as shaded intervals labeled at the family level.

In this study, “co-localization” refers to local genetic proximity on assembled short-read contigs or within ARG-centered windows, rather than reconstruction of complete plasmids, ICEs, transposons, or experimentally verified horizontal transfer events. Accordingly, mobility tiers were interpreted as a hierarchy of putative mobility-associated evidence, not as direct proof of transferability.

#### Taxonomic context analysis of ARG-carrying contigs

To provide a supplementary host-context prioritization layer, ARG-carrying contigs were taxonomically classified using Kraken2 with the full Standard database. Taxonomic assignments were parsed at the genus and species levels where available. Potential pathogen-associated ARG-carrying contigs were identified by matching genus- or species-level assignments to a curated list of taxa containing opportunistic, enteric, waterborne, or human-associated pathogenic members, including ESKAPE genera. Higher-rank assignments, ambiguous assignments, and unclassified contigs were not used to infer potential pathogen association. This analysis was interpreted as taxonomic-context prioritization of ARG-carrying contigs, rather than definitive host assignment or direct clinical risk estimation.

### Quantification and statistical analysis

All computations were carried out in R and Python unless explicitly stated. R analyses used vegan and related packages; Python analyses used SciPy, NumPy, pandas, and NetworkX. Sample sizes were SW, *n* = 11; RSD, *n* = 7; WT, *n* = 15; LS, *n* = 25; and HW, *n* = 5. Unless otherwise stated, n represents the number of independent environmental samples.

Bray-Curtis dissimilarities were calculated from transformed abundance matrices using log1p (RPM) where appropriate. Ordination was performed using principal coordinate analysis (PCoA), and habitat effects were evaluated by permutational multivariate analysis of variance (PERMANOVA) with 999 permutations. Group differences in total gene abundance were assessed using non-parametric Kruskal-Wallis tests, followed by multiple-comparison procedures when the omnibus test was significant. *p* values were adjusted using the Benjamini-Hochberg method.

Spearman correlations were calculated separately for ARG-MGE, ARG-BRG, and ARG-MRG pairs across samples using log1p-transformed RPM matrices. ARG nodes were defined at the feature, gene, or subtype level; MGE nodes were summarized at the family level; and BRG/MRG nodes followed the curated functional category scheme used for reads-level profiles. Significant associations were retained under |ρ| ≥0.60 with Benjamini-Hochberg false discovery rate (FDR) q < 0.05. To improve interpretability and comparability across networks while minimizing overplotting, a standardized subset of 220 edges per network was visualized, ranked by |ρ| after thresholding. When ties occurred near the cutoff, associations involving more recurrent and higher-connectivity nodes were retained to improve stability. Networks were visualized in Gephi v0.9.2. Network topology, including degree, betweenness, and closeness, was quantified in Python using NetworkX v3.3. Hub nodes were defined as the top 15 nodes ranked by degree in each network.

For contig-level enrichment analyses, including hub enrichment within module types and tier enrichment, Fisher’s exact tests were used with Benjamini-Hochberg correction. For boxplots, the center line indicates the median, boxes indicate the interquartile range, whiskers indicate 1.5 times the interquartile range, and points denote individual samples. Where significance asterisks are shown, ∗q < 0.05, ∗∗q < 0.01, and ∗∗∗q < 0.001.

## Supplemental information

Document S1. Figures S1–S13 and Tables S1–S3, S5, and S11–S13

Table S4. Human-health-oriented risk-rank annotation of detected drug-related ARG features

Table S6. Inventory of ARG-carrying contigs with MEF MGE calls, mobileOG mobility functions, and mobilization tier assignment

Table S7. Contig-level ARG-MGE proximity records on ARG-carrying contigs

Table S8. Contig-level ARG neighborhood records with BRG-like signals, including dual-function determinants

Table S9. Contig-level ARG neighborhood records with MRG-like signals, including dual-function determinants

Table S10. ARG-carrying contig taxonomic assignments and potential pathogen-associated taxonomic context
