# Supplementary material for: City-scale resistome-mobilome architecture and mobility-associated ARG backbones across a megacity watershed
Source: iScience. 2026 Jul 16;29(8):116841. doi: 10.1016/j.isci.2026.116841 (PMC13383857; doi:10.1016/j.isci.2026.116841)
Supplement: Document S1. Figures S1–S13 and Tables S1–S3, S5, and S11–S13 [file mmc1.pdf]

iScience, Volume *29*

Supplemental information

City-scale resistome-mobilome architecture and mobility-associated ARG backbones across a megacity watershed

Ningning Pi, Xiaoyao He, Lu Zhu, Xinjue Hou, Xiaoyan Wu, Juan Zhang, Lili Yang, Dongjun Shen, Zhifen Zou, Rong Xiang, and Xuan Wu

SUPPLEMENTAL FIGURES AND LEGENDS

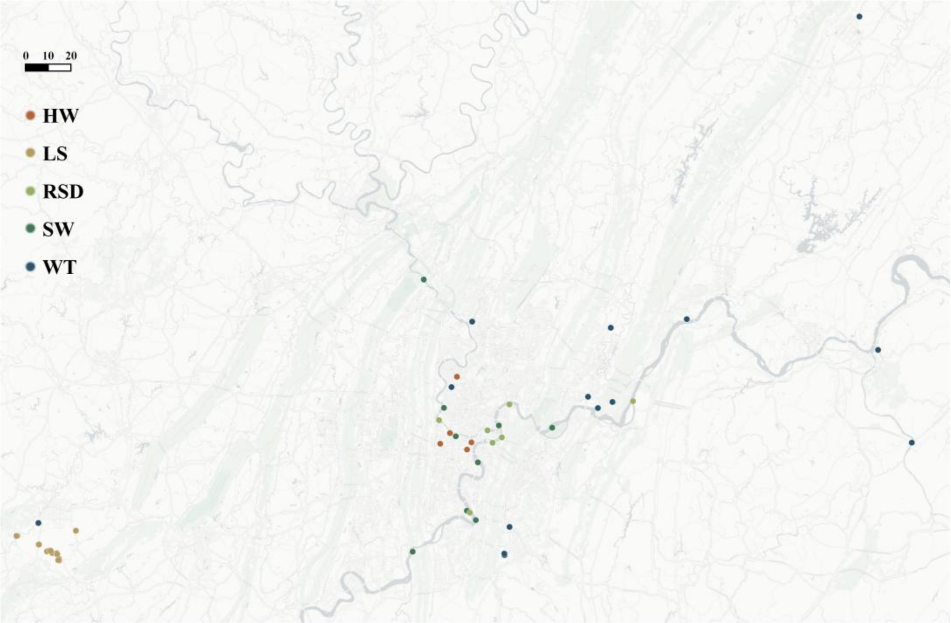

Fig. S1. Spatial distribution of sampling sites in Chongqing, China.

Sampling sites included surface water (SW), river sediment (RSD), wastewater treatment plant activated sludge (WT), livestock wastewater (LS), and hospital wastewater (HW). All sites were located within the Yangtze River watershed in Chongqing. Some points were slightly displaced for visualization purposes to avoid overlap.

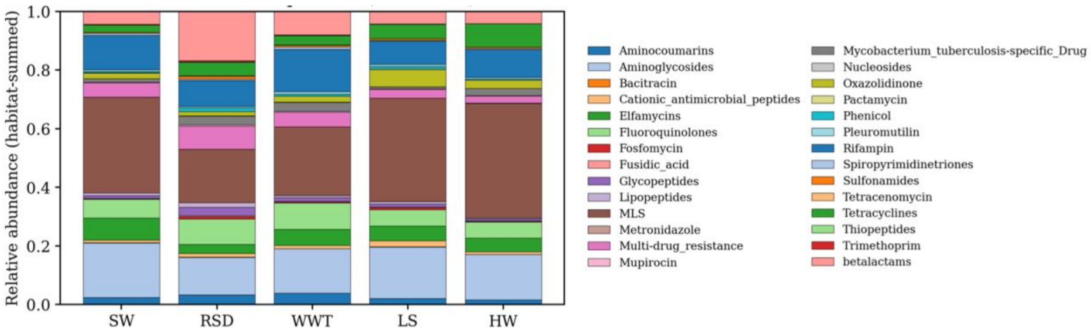

Figure S2. Full ARG class composition across habitats

Habitat-summed ARG class composition including all ARG classes. Stacked bars show relative abundance (habitat-summed RPM normalized to 1), without collapsing into a top-N set.

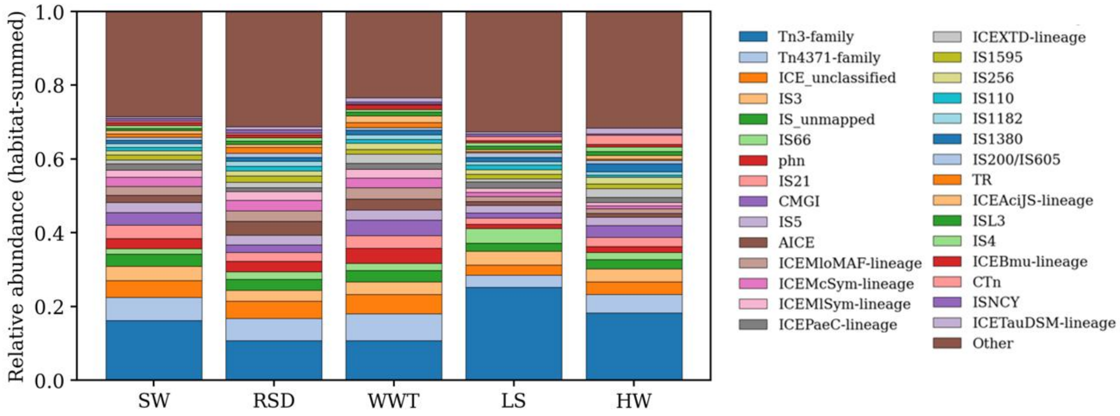

Figure S3. Extended MEF MGE family composition across habitats

Habitat-summed MEF MGE family composition showing the top 30 families plus "Other." Stacked bars represent habitat-summed RPM normalized to 1.

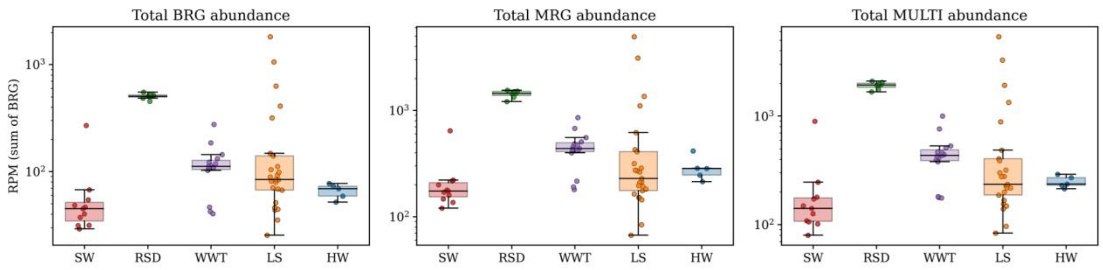

Figure S4. Total BRG, MRG, and MULTI abundances across habitats (reads level)

(A) Total BRG abundance (RPM; sum across BRG features per sample) by habitat.

(B) Total MRG abundance (RPM; sum across MRG features per sample) by habitat.

(C) Total MULTI abundance (RPM; sum across MULTI features per sample) by habitat.

For boxplots, the center line indicates the median, boxes indicate the interquartile range, whiskers indicate 1.5 times the interquartile range, and points denote individual samples.

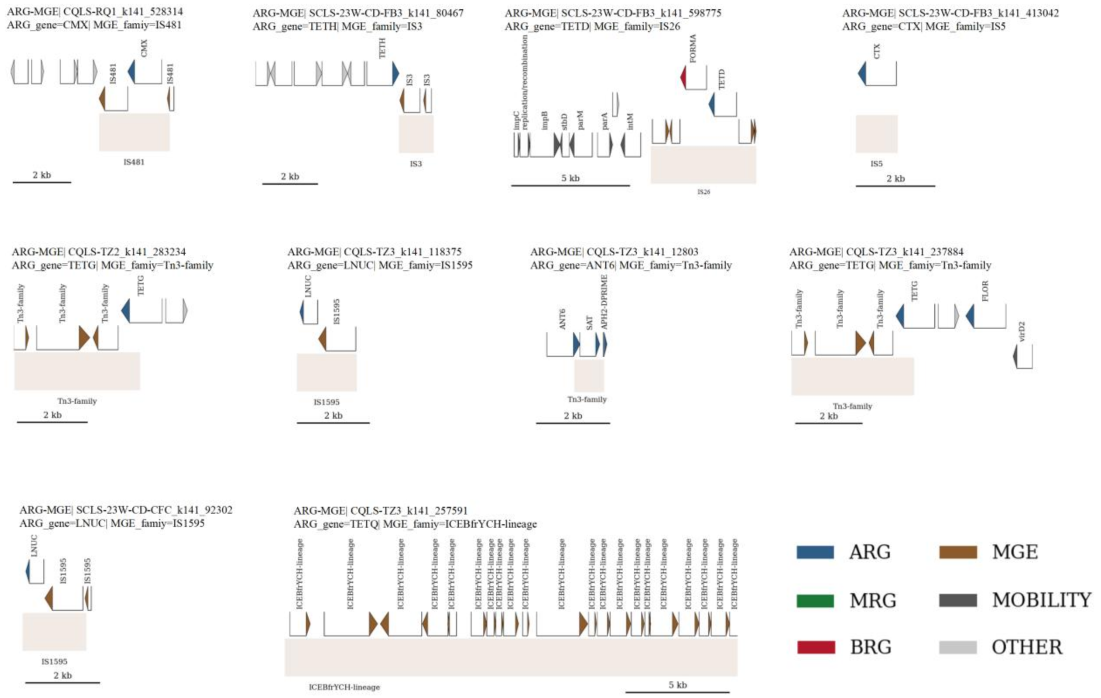

Figure S5. Additional ARG–MGE physical co-localization examples on ARG-carrying contigs

Additional contig neighborhood diagrams showing diverse ARG-MGE physical linkage patterns beyond the main Figure 4 panels. Colors and annotations follow Figure 4, with MEF MGE regions labeled at the family level.

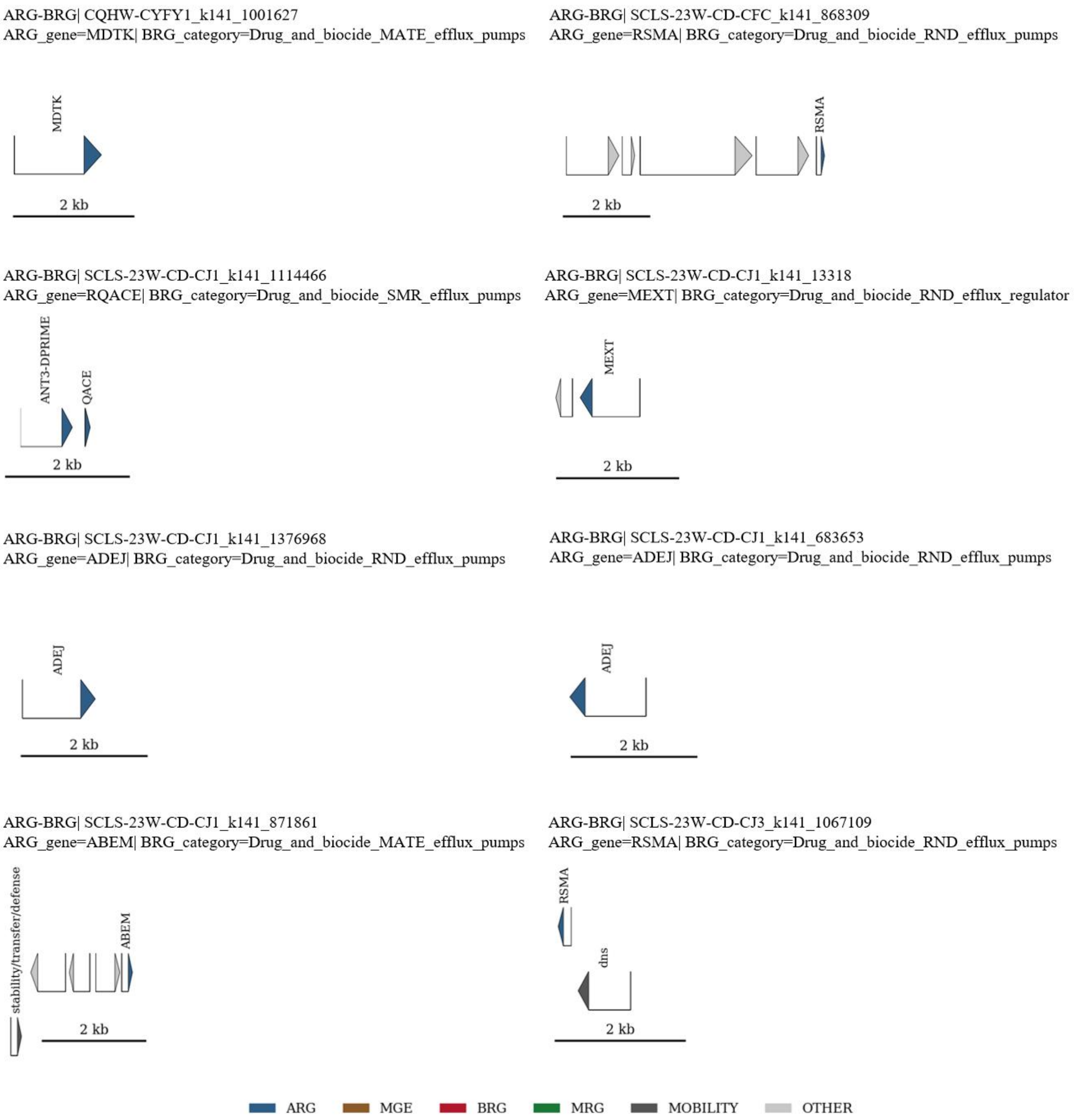

Figure S6. Additional ARG–BRG neighborhood examples on ARG-carrying contigs

Additional contig neighborhood diagrams illustrating ARG neighborhoods with BRG-like signals, including both adjacency to distinct BRG determinants and overlapping/dual-function efflux or regulatory ORFs (MinDist = 0). Colors and annotation conventions follow Figure 4.

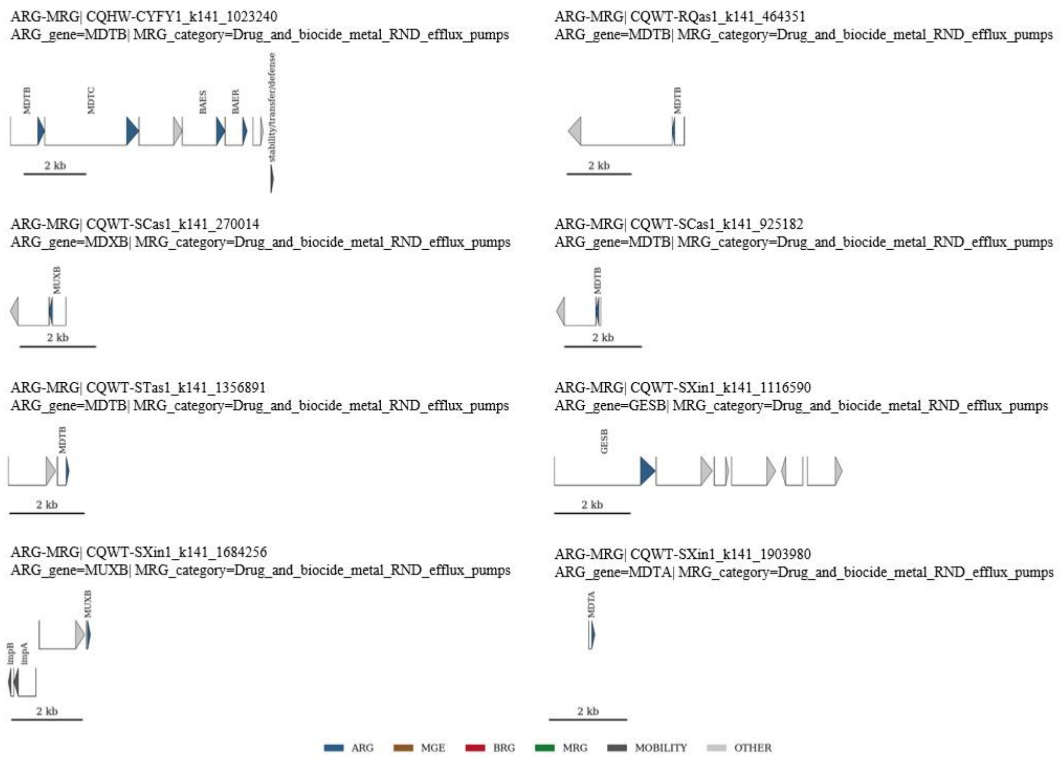

Figure S7. Additional ARG–MRG neighborhood examples on ARG-carrying contigs

Additional contig neighborhood diagrams illustrating ARG neighborhoods with MRG-like signals, including both adjacency to distinct MRG determinants and overlapping/dual-function efflux or regulatory ORFs (MinDist = 0). Colors and annotation conventions follow Figure 4.

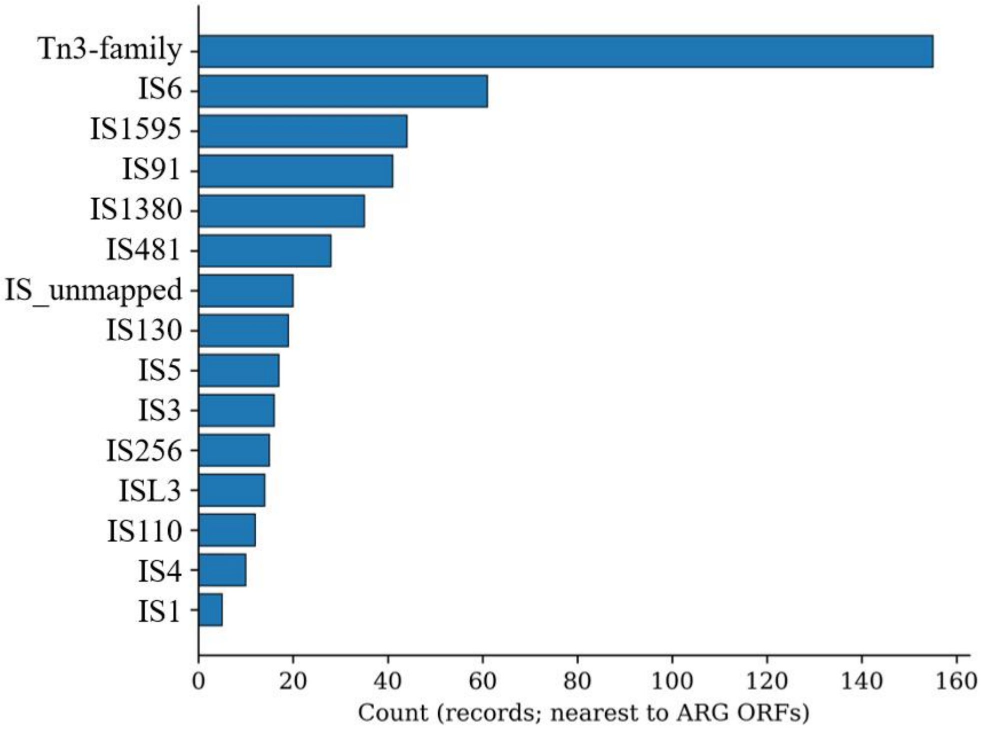

Figure S8. Top MEF MGE families across habitats

Top MGE family composition across habitats (top families shown explicitly; remaining families grouped as “Other”).

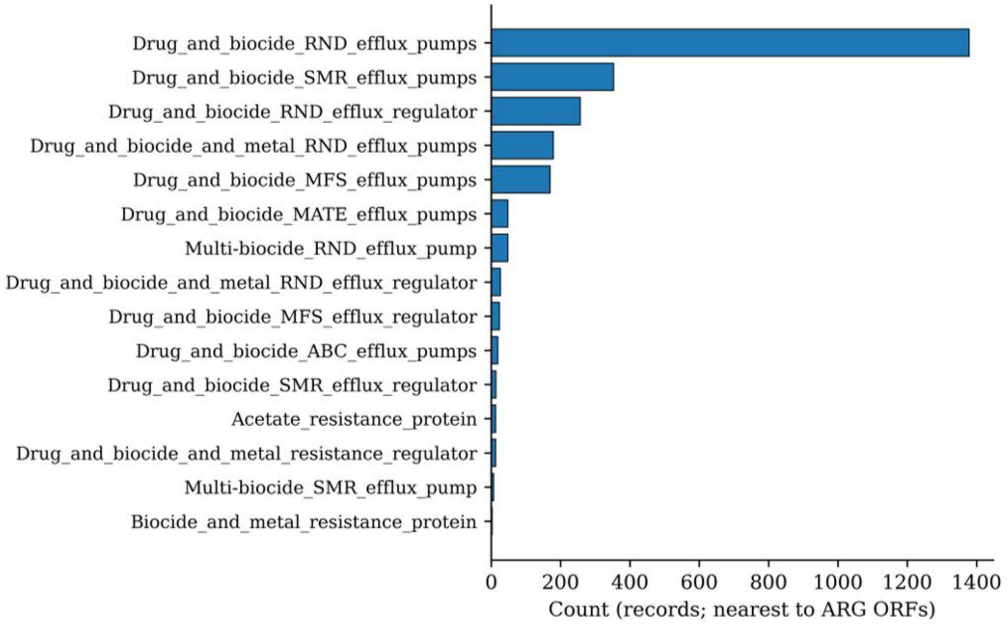

Figure S9. Top BRG categories across habitats

Top biocide-resistance determinant categories across habitats (top categories shown explicitly; remaining categories grouped as “Other”).

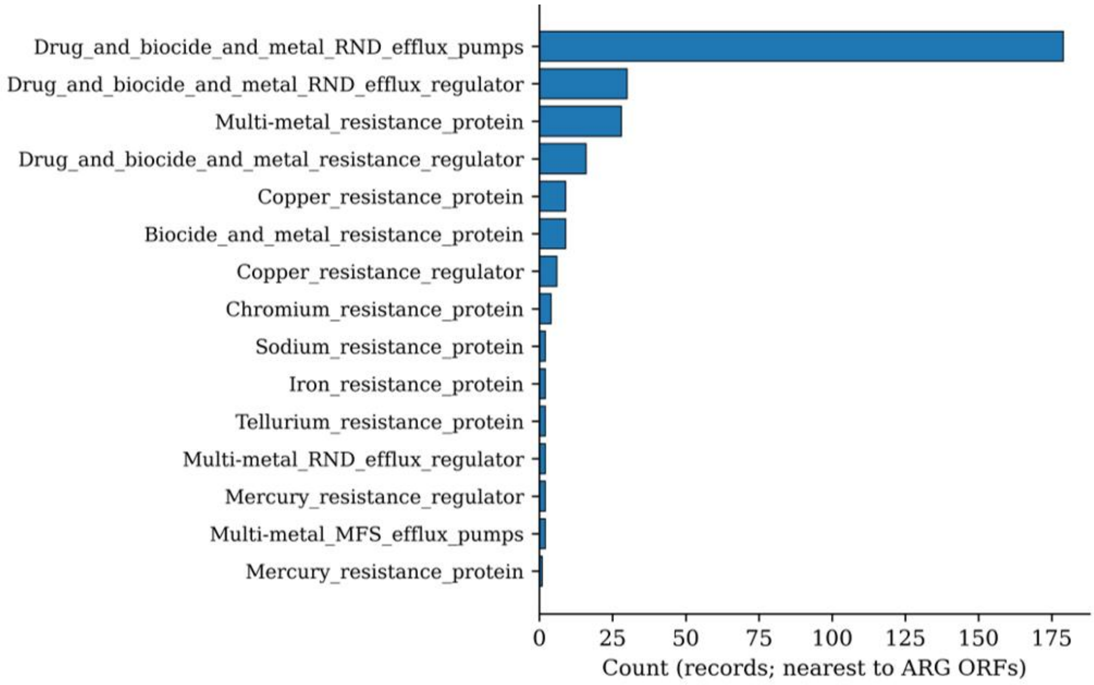

Figure S10. Top MRG categories across habitats

Top metal-resistance determinant categories across habitats (top categories shown explicitly; remaining categories grouped as “Other”).

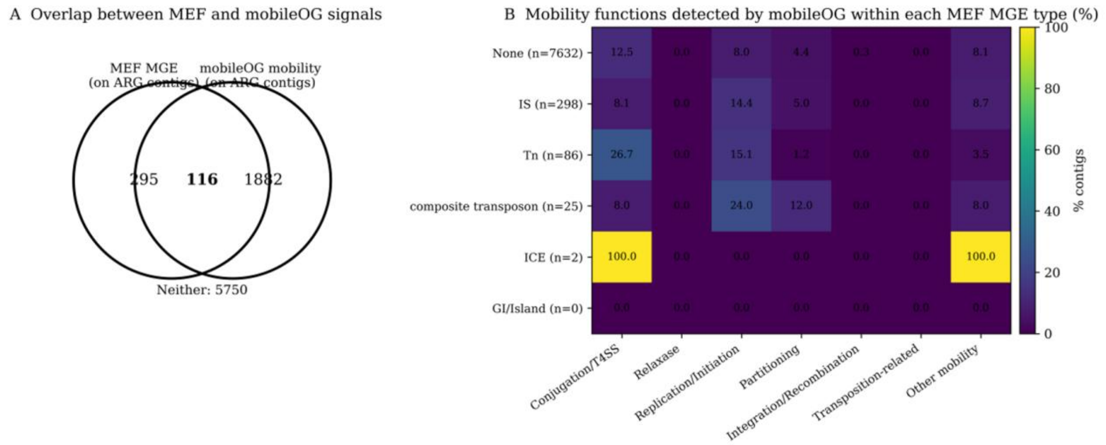

Figure S11. Complementarity between MEF element calls and mobileOG mobility functions on ARG-carrying contigs

(A) Venn diagram showing overlap between contigs with MEF-detected MGEs and contigs with mobileOG-annotated mobility ORFs among ARG-carrying contigs.

(B) Mobility-function composition (percent) detected by mobileOG among ARG-carrying contigs.

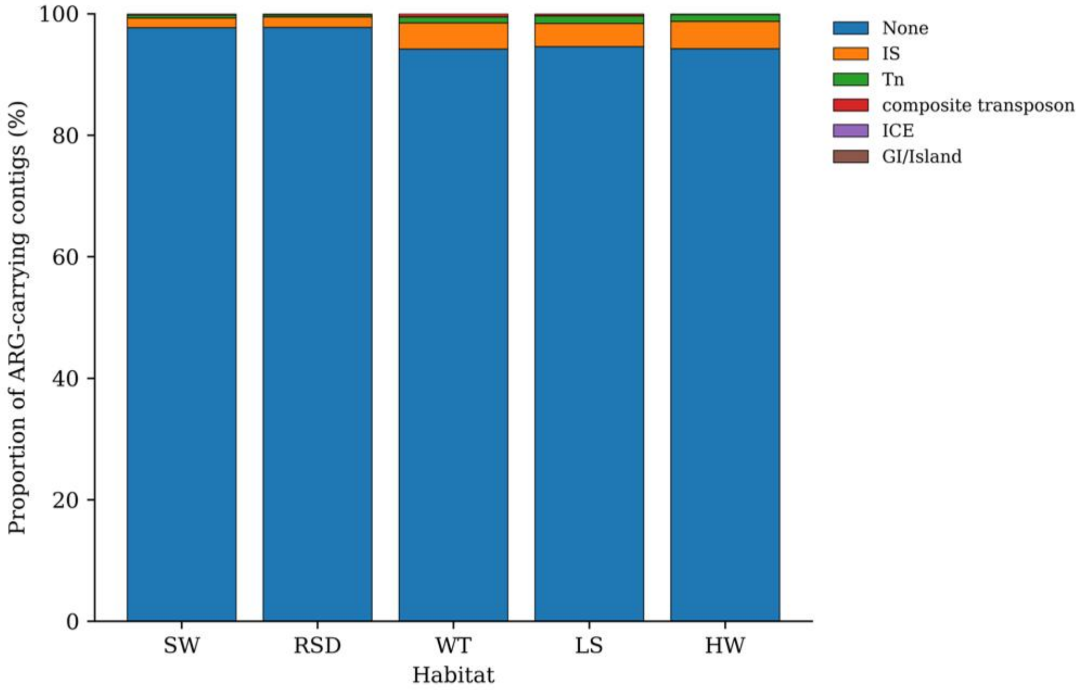

Figure S12. MEF-detected MGE types among ARG-carrying contigs by habitat

Stacked bars show the proportion of ARG-carrying contigs assigned to each MEF MGE type (None, IS, Tn, composite transposon, ICE, GI/Island) within each habitat.

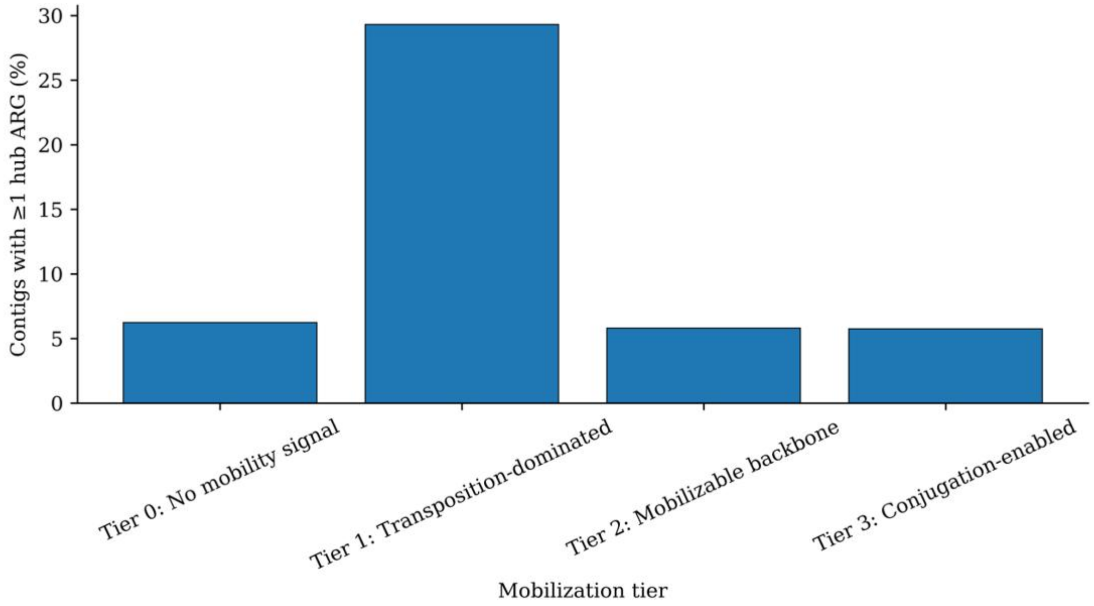

Figure S13. Hub ARG features are enriched on transposition-dominated contigs

Percentage of contigs containing ≥1 hub ARG feature across mobilization tiers (Tier 0–3). Hub ARGs are defined from reads-level correlation networks, and tiers follow the MEF–mobileOG stratification.

SUPPLEMENTAL TABLES

Table S4 and Tables S6–S10 are provided as separate Excel files because of their large size and/or machine-readable format.

Table S1. Kruskal-Wallis tests for differences in total ARG/MGE/BRG/MRG loads across habitats

Category	Kruskal_Wallis_H	p_value
ARG	30.4949	3.881E-06
MGE	29.2138	7.073E-06
BRG	25.3847	4.210E-05
MRG	25.6954	3.645E-05
MULTI	26.6057	2.388E-05

Table S2. Descriptive statistics of total RPM loads by habitat and category

Habitat	Category	n	mean_RPM	median_RPM	sd_RPM	IQR_RPM	min_RPM	max_RPM
SW	ARG	11	3047.2869	2913.7210	601.8739	403.7955	2592.6610	4748.0470
RSD	ARG	7	8716.0554	8432.4400	831.9366	311.6325	7825.1060	10470.3650
HW	ARG	5	5005.5534	5276.3300	1667.2523	2558.7420	2710.4000	6551.4590
LS	ARG	25	5646.9738	4595.5620	2524.6656	3388.2510	2323.0070	12921.9730
WWT	ARG	15	3164.7505	2859.4760	1206.7652	691.6835	1924.4170	6875.4350
SW	MGE	11	7780.1997	5952.6340	4792.7907	2088.7855	5116.0560	21626.8950
RSD	MGE	7	55116.6054	55800.8800	2038.1400	2015.8015	52230.6610	57689.6760
HW	MGE	5	12809.1534	11849.7880	3096.0296	2512.8700	9442.4840	17586.9610
LS	MGE	25	14786.8674	11798.4180	7076.1808	7853.7960	5838.4320	33418.2930
WWT	MGE	15	14317.9501	13946.9780	6394.8143	2997.4995	5501.3950	31159.2670
SW	BRG	11	63.6922	45.0730	69.0728	17.0320	29.0950	269.1270
RSD	BRG	7	506.4489	506.3500	31.2735	24.8975	453.7890	553.5700
HW	BRG	5	65.9078	68.9880	10.4355	13.8220	51.7110	77.2660
LS	BRG	25	232.0959	83.9610	403.4299	72.8110	25.4340	1824.8590
WWT	BRG	15	117.3119	111.7700	58.3438	22.8445	40.3620	275.2800
SW	MRG	11	215.3540	174.3420	146.0294	55.3845	120.3890	645.1830
RSD	MRG	7	1427.1057	1441.4110	119.0578	130.6535	1212.4340	1549.1660
HW	MRG	5	289.0882	284.4490	76.2632	39.2190	213.6040	414.5750
LS	MRG	25	621.4471	228.7540	1098.3810	231.8180	67.0740	4936.7550
WWT	MRG	15	446.9997	436.8980	174.9981	86.5430	179.9910	853.3270
SW	MULTI	11	209.5639	141.0210	232.1397	68.5120	80.0110	895.4950
RSD	MULTI	7	1913.5950	1937.7900	152.6391	166.9165	1670.4690	2099.6730
HW	MULTI	5	248.3458	236.5530	31.5995	40.1140	213.7180	291.3040
LS	MULTI	25	702.9940	235.6680	1208.8097	216.6290	83.5990	5393.8400
WWT	MULTI	15	450.9739	435.3630	214.3031	101.6790	176.6550	1001.2700

Table S3. PERMANOVA results for habitat effects on resistome and mobilome composition (Bray-Curtis, log1p RPM)

Category	Feature_level	Transform	Distance	pseudo_F	R^2	p_value	df_between	df_within	permutations
ARG	Subtype	log1p(RPM)	Bray-Curtis	16.3908	0.5306	0.001	4	58	999
MGE	Family	log1p(RPM)	Bray-Curtis	19.3350	0.5714	0.001	4	58	999
BRG	Feature	log1p(RPM)	Bray-Curtis	9.5409	0.3969	0.001	4	58	999
MRG	Feature	log1p(RPM)	Bray-Curtis	10.1189	0.4110	0.001	4	58	999
MULTI	Feature	log1p(RPM)	Bray-Curtis	10.2001	0.4130	0.001	4	58	999

Table S5 Spearman correlations between total ARG load and MGE_BRG(+MULTI)_MRG(+MULTI) axes (overall and by habitat)

Group	Axis	n	Spearman_rho	p_value	q_value
All	MGE	63	0.8000	3.687E-15	1.106E-14
All	BRG (+MULTI)	63	0.5828	5.399E-07	8.099E-07
All	MRG (+MULTI)	63	0.5412	4.648E-06	4.648E-06
HW	MGE	5	0.7000	1.881E-01	4.271E-01
HW	BRG (+MULTI)	5	-0.1000	8.729E-01	8.729E-01
HW	MRG (+MULTI)	5	-0.6000	2.848E-01	4.271E-01
LS	MGE	25	0.9031	6.523E-10	1.957E-09
LS	BRG (+MULTI)	25	0.4908	1.274E-02	1.911E-02
LS	MRG (+MULTI)	25	0.4508	2.373E-02	2.373E-02
RSD	MGE	7	0.3214	4.821E-01	8.192E-01
RSD	BRG (+MULTI)	7	0.1071	8.192E-01	8.192E-01
RSD	MRG (+MULTI)	7	0.1071	8.192E-01	8.192E-01
SW	MGE	11	0.5818	6.042E-02	1.420E-01
SW	BRG (+MULTI)	11	0.4727	1.420E-01	1.420E-01
SW	MRG (+MULTI)	11	0.5273	9.557E-02	1.420E-01
WT	MGE	15	0.8679	2.752E-05	2.752E-05
WT	BRG (+MULTI)	15	0.8857	1.120E-05	1.680E-05
WT	MRG (+MULTI)	15	0.9429	1.425E-07	4.274E-07

Table S11 Mobilization tier composition within mutually exclusive co-selection module classes

Module_type	Mobilization_tier	n	pct_within_module
ARG only	Tier 0: No mobility signal	4288	71.9705
ARG only	Tier 1: Transposition-dominated	300	5.0352
ARG only	Tier 2: Mobilizable backbone	582	9.7684
ARG only	Tier 3: Conjugation-enabled	788	13.2259
ARG+BRG	Tier 0: No mobility signal	1637	86.3852
ARG+BRG	Tier 1: Transposition-dominated	6	0.3166
ARG+BRG	Tier 2: Mobilizable backbone	68	3.5884
ARG+BRG	Tier 3: Conjugation-enabled	184	9.7098
ARG+MRG	Tier 0: No mobility signal	1	33.3333
ARG+MRG	Tier 1: Transposition-dominated	1	33.3333
ARG+MRG	Tier 2: Mobilizable backbone	1	33.3333
ARG+BRG+MRG (composite)	Tier 0: No mobility signal	148	79.1444
ARG+BRG+MRG (composite)	Tier 2: Mobilizable backbone	4	2.1390
ARG+BRG+MRG (composite)	Tier 3: Conjugation-enabled	35	18.7166

Table S12 Tier 3 enrichment_depletion across module groups (Fisher’s exact tests)

Group	Outcome	a	b	c	d	OR	PValue
Composite modules	Tier3	35	152	972	6884	1.6308	0.0135
ARG+BRG modules	Tier3	219	1863	788	5173	0.7717	0.0012
ARG+MRG modules	Tier3	35	155	972	6881	1.5985	0.0191

Table S13 Enrichment of hub ARG features within co-selection module classes (Fisher's exact test)

Module_type	n contigs	n with hub	pct with hub	Fisher OR composite vs others	Fisher pvalue composite vs others
ARG only	5958	502	8.4256	2.7247	6.530E-06
ARG+BRG	1895	31	1.6359	2.7247	6.530E-06
ARG+BRG+MRG (composite)	187	31	16.5775	2.7247	6.530E-06
ARG+MRG	3	1	33.3333	2.7247	6.530E-06
